# Association between the COVID-19 infodemic and depression symptom screening in older adults*

**DOI:** 10.1590/1518-8345.7454.4461

**Published:** 2025-05-19

**Authors:** Patricia Rodrigues Braz, Tiago Ricardo Moreira, Andréia Queiroz Ribeiro, Rosimere Ferreira Santana, Jack Roberto Silva Fhon, Alexandre Favero Bulgarelli, Vilanice Alves de Araújo Püschel, Edna Aparecida Barbosa de Castro, Denise Rocha Raimundo Leone, Ricardo Bezerra Cavalcante

**Affiliations:** 1 Universidade Federal de Juiz de Fora, Juiz de Fora, MG, Brazil; 2 Universidade Federal de Viçosa, Viçosa, MG, Brazil; 3 Universidade Federal Fluminense, Niterói, RJ, Brazil; 4 Universidade de São Paulo, Escola de Enfermagem, São Paulo, SP, Brazil; 5 Universidade Federal do Rio Grande do Sul, Porto Alegre, RS, Brazil

**Keywords:** Infodemic, COVID-19, Health of the Elderly, Mental Health, Depression, Depressive Disorder

## Abstract

to analyze the profile of exposure to COVID-19 information and its association with depressive symptom screening in a sample of older adults in Brazil.

cross-sectional study using data collected through a web-based survey with 3,307 participants recruited via social media and email. Descriptive and bivariate analyses were conducted to estimate associations of interest, as well as crude and adjusted logistic regression, controlling for predictive, sociodemographic and infodemic variables.

a significant association was found between the presence of depression symptoms among older adults who were exposed to social media and television for three to six hours or more and those who reported not having been exposed to any news and information about COVID-19 on television.

older women frequently exposed to COVID-19-related information on television and social media for two- to six-hour periods showed depression symptoms. This study contributes to research on infodemics and mental health by addressing a research gap on the relationship between depressive symptom screening and the profile of exposure to COVID-19 information in a sample of older adults.

## Highlights

**(1)** There is an association between infodemic variables and depression screening. **(2)** Innovation in research on the impacts of the infodemic on depression screening. **(3)** Older adults who were not exposed to the news also showed signs of depression. **(4)** The study provides contributions to research on infodemics and mental health.

## Introduction

COVID-19 was considered a new type of viral condition, and at the time of its outbreak, scientific information and insights from governmental institutions about the disease were still scarce. In this context, there was an excessive spread of information by the media. This phenomenon was characterized as an infodemic, meaning an overload of information about a specific topic, which can exponentially multiply in a short period of time due to a specific event^(1-1)^. An infodemic also fosters the emergence of incorrect information, misinformation and the manipulation of information with questionable intent^(2)^. The COVID-19 infodemic was widely recognized for aggravating the pandemic’s challenges, acting as a catalyst for the spread of inappropriate content^(3)^.

Older adults, especially in Brazil, may be more vulnerable to misinformation and, consequently, to contributing to the spread of fake news. This is not necessarily due to age itself but to certain traits that are more common in this stage of life.

A number of factors seems to make older adults in Brazil more susceptible to consuming and spreading fake news about COVID-19, one of them being functional illiteracy. According to the most recent survey on functional illiteracy in Brazil, from 2018, 10.3% of the population aged 60 and older were found to be functionally illiterate^(4)^. In addition, there are weaknesses in health literacy, digital literacy, media literacy, and scientific literacy^(5-5)^.

This infodemic context has negatively affected the mental health of older adults. Studies^(8-8)^ carried out with adult samples identified an increase in depression cases associated with information overload, as well as recurring depression triggered by negative information about COVID-19^(13-13)^. Moreover, there is evidence that individuals with depression tend to use social media frequently^(15)^.

During the COVID-19 pandemic, there was a significant rise in depression diagnoses worldwide^(16-16)^. In the first year of the pandemic, the global prevalence of depression increased by 25%. Before the pandemic, in early 2020, approximately 193 million people were diagnosed with major depressive disorder. After the onset of the pandemic, estimates show a surge to 246 million cases of major depressive disorder, a 28% increase in 2021^(17)^. Currently, Brazil has the highest prevalence of depression in Latin America and the second highest in the Americas^(18)^, with older adults being most affected^(19)^.

According to the International Classification of Diseases (ICD-10), the diagnostic criteria for a depressive episode include low mood, reduced energy, decreased activity, impaired ability to experience pleasure, loss of interest, reduced concentration and fatigue. In mild depressive episodes, at least two or three of these symptoms are generally present. In severe depressive episodes without psychotic symptoms, several symptoms are more pronounced and distressing, often involving loss of self-esteem and feelings of guilt. In addition, suicidal ideation is common and a range of somatic symptoms is generally observed^(20)^.

Given the above, studies show that the COVID-19 infodemic has negatively impacted the mental health of older adults. Therefore, it is important to investigate this phenomenon and its consequences on the mental health of older people, especially regarding depression symptom screening. Research on this subject with older adults remains scarce. This study aimed to analyze the profile of exposure to COVID-19 information and its association with depression symptom screening in a sample of older adults in Brazil.

## Method

### Study design and period

This is a descriptive, exploratory and cross-sectional study carried out with older adults in Brazil. Data were collected between July 2020 and March 2021.

### Sample and selection criteria

The sample size for this multicenter study was estimated for each municipality, considering the local population of older adults, by using the formula: n= N. Z².p.(1−p) /Z².p.(1−p). + e². (N−1), in which “n” is the calculated sample size, “N” is the population size, “Z” is the standard normal variable associated with the confidence level (in this case, 95%), “p” is the true probability of the event (P = (1−P) = 0.5, assuming maximum probability), and “e” is the sampling error, set at 5%. For partial data, the sample considered a 50% prevalence of the phenomenon, a 3% error margin, a 95% confidence level +10% for multiple analyses and +20% to account for potential losses within the sample (unanswered questions). Non-probabilistic sampling was used in Brazil. The initial database included 3,321 participants. However, duplicates and incomplete responses were excluded, resulting in a final sample of 3,307 Brazilian older adults who took part in the study.

Eligible participants were Brazilian older adults aged 60 or above, with access to social media, email and/or telephone, and the ability to take the survey via social media or phone. The exclusion criteria were individuals who were unable to answer the questionnaire via digital media or phone and residents of Long-Term Care Institutions for Older Adults (LTCIs), considering that the actual process of institutionalizing older adults may contribute to the development or worsening of depressive and anxiety disorders^(21)^.

Different strategies were used to ensure the representativeness of the sample. Online snowball sampling was employed by sharing the survey link with geriatrics and gerontology scientific societies, healthcare centers, retiree associations and, directly, older adults already involved in research and outreach activities at the collaborating research centers.

### Data collection instruments and study variables

The data collection instrument for exposure to COVID-19 information was adapted from previous studies on infodemics^(8-8)^. Besides socioeconomic variables, it included variables related to exposure to the COVID-19 infodemic on social media, the radio and television, focusing on the type of medium accessed and the duration of exposure (frequency and hours).

The Geriatric Depression Scale (GDS) was used to evaluate depression symptom screening, adopting the 5/6 cutoff point (non-case/case), which is considered appropriate for screening, enabling early identification and categorizing depression symptoms in older adults in non-specialized settings in Brazil^(23)^.

### Data collection

The data collection instrument was set up as a web-based survey. Upon first accessing the link, older adults were directed to a digital Informed Consent Form (ICF) to read and choose whether to accept or decline participation in the study. Their decision to participate or not was automatically recorded in the database generated by the web-based survey. Those who chose to proceed were given access to the survey questions.

### Data treatment and analysis

Data treatment and analysis were performed using the Statistical Package for the Social Sciences® (SPSS) version 23.0. Descriptive analyses were conducted by characterizing the demographics of the participants and the variables related to exposure to news and information about COVID-19 on the media. For qualitative variables, absolute and relative frequencies were estimated. For quantitative variables, measures of central tendency (mean and median) and dispersion (standard deviation, interquartile range, minimum and maximum) were computed, depending on the data distribution (whether symmetric or asymmetric).

The association between infodemic variables and outcomes of depression symptom screening was assessed using the chi-square test. Subsequently, multiple logistic regression analyses were conducted, both crude and adjusted (by gender, age group, education level, shared housing and income changes during the pandemic), to examine the association between the independent variables of interest and the outcome for depression, with respective 95% confidence intervals. The significance level adopted for all tests was 5%.

### Ethical aspects

The study was approved by the Brazilian National Commission for Research Ethics (Conep) on July 3, 2020 – Ethical Approval Certificate (CAAE): 31932620.1.1001.5147, under Opinion No. 4.134.050. Data collection began following approval. All ethical and legal requirements for research involving humans were met, in accordance with the regulatory provisions of Resolution No. 466/12 of the Brazilian National Health Council.

Participants were assigned numerical identifiers to assure anonymity during the study and were informed about its objective, rationale and procedures. They were also informed of their voluntary participation, with no financial benefits or costs, and that the findings would only be disclosed at events and/or in scientific journals.

## Results

A total of 3,307 older adults took part in the study, of whom 68.4% were women, 38.9% were aged between 60 and 64, 55.5% were married and 71.5% self-identified as White. Regarding residence, 95.6% lived in urban areas and 57.0% shared the household with one or two people. Among the participants, only 8.9% had not attended or completed elementary education, while 19.5% had completed higher education. The majority (40.6%) used both free and paid healthcare, and 73.8% reported that the pandemic had not affected their monthly income.

Regarding screening for depression symptoms related to the sociodemographic variables, it was found that 66.7% of older adults who preferred not to declare their gender presented depression symptoms. Among those aged 80 or older, 44.7% presented depression symptoms, the age group with the highest percentage. Regarding marital status, 46.3% of single older adults presented depression symptoms. As for shared housing, 37.3% of those living alone showed depression symptoms. In terms of education level, 47.1% of older adults who had not had the opportunity to study or complete elementary education presented depression symptoms (Table 1).

**Table 1 -:** Demographic profile and depression symptom screening in older adults participating in the study (n = 3307). Brazil, 2021

**Parameter**	**N**	**%**	**Depression symptom screening**			
			**Non-case**		**Case**	
			**N**	**%**	**N**	**%**
**Gender**						
Man	2,250	68.4	1347	59.9%	903	40.1%
Woman	1,039	31.6	638	61.4%	401	38.6%
Not declared	18	0.5	6	33.3%	12	66.7%
**Age group (years)**						
60 to 64	1,285	38.9	799	62.2%	486	37.8%
65 to 69	921	27.9	543	59.0%	378	41.0%
70 to 74	503	15.2	309	61.4%	194	38.6%
75 to 79	334	10.1	194	58.1%	140	41.9%
80 and above	264	8.0	146	55.3%	118	44.7%
**Marital status**						
Married/cohabiting	1,835	55.5	1119	61.0%	716	39.0%
Widowed	598	18.1	340	56.9%	258	43.1%
Separated/divorced	509	15.4	336	66.0%	173	34.0%
Single	365	11.0	196	53.7%	169	46.3%
**Ethnicity/skin color**						
White	2,364	71.5	1436	60.7%	928	39.3%
Non-White	943	28.5	555	58.9%	388	41.1%
**Shared housing**						
Lives alone	587	17.8	368	62.7%	219	37.3%
One or more people	1,886	57.0	1129	59.9%	757	40.1%
Three or more people	834	25.2	494	59.2%	340	40.8%
**Homeowner**						
Yes	2,756	83.3	1668	60.5%	1088	39.5%
No	551	16.7	323	58.6%	228	41.4%
**Residence area**						
Urban	3,160	95.6	1910	60.4%	1250	39.6%
Rural	147	4.4	81	55.1%	66	44.9%
**Education level**						
None or incomplete elementary	295	8.9	156	52.9%	139	47.1%
Elementary	713	21.6	409	57.4%	304	42.6%
Secondary	718	21.7	449	62.5%	269	37.5%
Higher	645	19.5	389	60.3%	256	39.7%
Specialist degree	512	15.5	322	62.9%	190	37.1%
Master’s or doctoral degree	424	12.8	266	62.7%	158	37.3%
**Healthcare used**						
Paid only (including health insurance)	1,133	34.3	692	61.1%	441	38.9%
Both (free and paid)	1,343	40.6	823	61.3%	520	38.7%
Free only	814	24.6	466	57.2%	348	42.8%
None	17	0.5	10	58.8%	7	41.2%
**Pension**						
Yes	2,565	77.6	1520	59.3%	1045	40.7%
No	740	22.40	470	63.5%	270	36.5%
**Income altered by pandemic**						
No	2,437	73.8	1469	60.3%	968	39.7%
Yes, reduced	787	23.8	47	58.8%	33	41.2%
Yes, increased	80	2.4	474	60.2%	313	39.8%

Regarding the most commonly used sources to access news and information about COVID-19 during the day, 81.1% of older adults used television, 58.8% social media and only 26.48% used the radio. The older adults also reported perceiving the exposure to COVID-19 news and information over a one-week period (seven days), ranging from “none” to “frequent.” Television was reported to be a “frequent” source of exposure by 44.5%, followed by social media, mentioned as “occasional” (44.3%). In contrast, the radio did not represent a source of exposure for the majority of older adults (59.1%). Regarding hours of exposure, 39.3% of older adults were exposed to television for three hours or more, whereas 32.8% accessed social media for two to five hours, and 37.0% were exposed to the radio for one hour or more (Table 2).

**Table 2 -:** Characterization of sources of information most commonly used to access news and updates about COVID-19 among the older adult participants (n = 3307). Brazil, 2021

**Sources of information**	**N**	**%**
**Social media (N=3,303)**		
Yes	1,943	58.8
No	1,361	41.2
**Frequency of exposure**		
None	822	24.9
Occasional	1,464	44.3
Frequent	1,021	30.9
**Hours of exposure to news and information about COVID-19 on social media**		
Zero	848	25.6
One	811	24.5
Two to five	1084	32.8
Six or more	560	16.9
**Television (N=3,304)**		
Yes	2,680	81.1
No	624	18.9
**Frequency of exposure**		
None	394	11.9
Occasional	1,440	43.5
Frequent	1,473	44.5
**Hours of exposure to news and information about COVID-19 on television**		
Zero	431	13.0
One	884	26.7
Two	685	20.7
Three or more	1301	39.3
**Radio (N=3,304)**		
Yes	876	26.5
No	2,429	73.5
**Frequency of exposure**		
None	1,956	59.1
Occasional	956	28.9
Frequent	395	11.9
**Hours of exposure to news and information about COVID-19 on the radio**		
Zero	2083	63.0
One or more	1223	37.0

A significant association (*P* > 0.05, meaning a probability greater than 5%) was found between the outcome of depression symptom screening and the variables of frequency of exposure to television (*P* = 0.01) and hours of exposure to COVID-19 news and information on social media (*P* = 0.00) and television (*P* = 0.02). Regarding the variable of frequency of exposure to television, 42.9% of older adults who reported no exposure to information presented depression symptoms. As for the hours of exposure, 44.3% of older adults who reported being exposed to COVID-19 news and information on social media for six hours or more presented depression symptoms. Similarly, 43.2% of those who reported being exposed for three hours or more to COVID-19 news and information on television also presented depression symptoms (Table 3).

The analysis showed that, in the crude logistic regression, the following variables were associated with the presence of depression symptoms: frequent exposure to COVID-19 news and information on the radio (*P* = 0.02) and television (*P* = 0.00), exposure for two hours (*P* = 0.00) or three hours or more (*P* = 0.04) on television, and exposure for two to five hours (*P* = 0.00) or six hours or more (*P* = 0.02) on social media. In turn, in the adjusted analysis, the infodemic variables that remained associated with the presence of depression symptoms (*P* ≤ 0.05) were: frequent exposure to television and the radio, exposure for two to five hours, or six hours or more on social media, and exposure for two hours on television (Table 4).

**Table 3 -:** Association between infodemic variables and depression symptom screening among older adult participants (n = 3307). Brazil, 2021

**Parameter**	**Presence of depression symptoms**				***P* Value**
	**Non-case**		**Case**		
	**N**	**%**	**N**	**%**	
**Frequency of exposure to social media**					0.08
None	463	56.3%	359	43.7%	
Occasional	920	62.8%	544	37.2%	
Frequent	608	59.5%	413	40.5%	
**Frequency of exposure to television**					0.01
None	225	57.1%	169	42.9%	
Occasional	909	63.1%	531	36.9%	
Frequent	857	58.2%	616	41.8%	
**Frequency of exposure to the radio**					0.07
None	1182	60.4%	774	39.6%	
Occasional	591	61.8%	365	38.2%	
Frequent	218	55.2%	177	44.8%	
**Hours of exposure to news and information about COVID-19 on social media**					0.00
Zero	482	56.8%	366	43.2%	
One	529	65.2%	282	34.8%	
Two to five	665	61.3%	419	38.7%	
Six or more	312	55.7%	248	44.3%	
**Hours of exposure to news and information about COVID-19 on television**					0.02
Zero	254	58.9%	177	41.1%	
One	573	64.8%	311	35.2%	
Two	421	61.5%	264	38.5%	
Three or more	739	56.8%	562	43.2%	
**Hours of exposure to news and information about COVID-19 on the radio**					0.83
Zero	1251	60.1%	832	39.9%	
One or two	739	60.4%	484	39.6%	

**Table 4 -:** Crude and adjusted logistic regression model for the total score of the Geriatric Depression Scale administered to the older adult participants (n = 3307). Brazil, 2021

**Presence of depression symptoms (logistic regression)**		
	Crude Analysis OR(95%CI)*	Adjusted Analysis OR(95%CI)*
**Frequency of exposure to social media**		
None	1	1
Occasional	1.14 (0.94 – 1.37)	1.01 (0.82 – 1.25)
Frequent	0.87 (0.73 – 1.02)	0.85 (0.72 – 1.01)
**Frequency of exposure to television**		
None	1	1
Occasional	1.04 (0.83– 1.30)	1.06 (0.85 – 1.34)
Frequent	0.81 (0.70 –0.94)	0.81 (0.70 – 0.94)
**Frequency of exposure to the radio**		
None	1	1
Occasional	0.80 (0.64 – 1.00)	0.80 (0.64 – 1.00)
Frequent	0.76 (0.60 – 0.96)	0.74 (0.58 – 0.95)
**Hours of exposure to news and information about COVID-19 on social media**		
Zero	1	1
One	0.95 (0.77 – 1.18)	0.86 (0.68 – 1.09)
Two to five	0.67 (0.53 – 0.83)	0.68 (0.54 – 0.85)
Six or more	0.79 (0.64 – 0.97)	0.80 (0.64 – 0.98)
**Hours of exposure to news and information about COVID-19 on television**		
Zero	1	1
One	0.91 (0.73 – 1.14)	0.95 (0.76 – 1.20)
Two	0.71 (0.59 – 0.85)	0.73 (0.61 – 0.87)
Three or more	0.82 (0.68 – 0.99)	0.85 (0.70 – 1.02)
**Hours of exposure to news and information about COVID-19 on the radio**		
Zero	1	1
One or two	1.01 (0.87 – 1.17)	1.03 (0.89 – 1.20)

*OR(95%CI) = Odds ratio and confidence interval

## Discussion

The study aimed to investigate the COVID-19 infodemic and its consequences on the mental health of older adults by screening for the presence of depression symptoms.

Regarding the infodemic variables, the time frame of the pandemic proves to be relevant. During the study period, from July 2020 to March 2021, the COVID-19 epidemiological situation was alarming. According to the Brazilian Ministry of Health^(24)^, the average number of deaths was approximately 1,026 in the second half of 2020; the document also shows that March 2021 had the highest percentage of deaths, with an average of 2,207 deaths per day^(24)^.

Social distancing rules were still in effect, leading to increased media consumption and television viewing among the adult population. Information on the number of deaths and people infected was prominently featured in the media, as well as information related to the political dispute over the purchase and distribution of vaccines, as the first vaccines only arrived in the country in January 2021^(8,8)^.

The results of this survey identified that television was the most commonly used medium for accessing information and was also cited as the source with the highest frequency of exposure among older adults, lasting three hours or more, followed by social media. The radio, on the other hand, was the least accessed source of information.

A study conducted in Brazil during the COVID-19 pandemic with 45,161 individuals, 20.3% of whom were older adults aged 60 or more, found an increase in television viewing, with older adults being the group with the highest average time spent in front of the television. The study also reported that 61.6% of the participants were exposed to this medium for three hours or more^(25)^.

Another investigation analyzed the information-seeking behavior of older adults during the COVID-19 pandemic and found that television was the main news source cited by them^(26)^. The older adults who were interviewed mentioned that their preference for television was because it provided easily available and fast information, with updates on the daily events happening worldwide^(27)^.

In the case of older adults, television remains the simplest, fastest and easiest way to access information^(3,12,26)^, as they may face greater difficulty using and handling computers and mobile phones.

Another study conducted with 799 adult participants in Rio Grande do Sul found that the most commonly used medium to access information about COVID-19 was television (76.3%), followed by social media, especially Facebook (37%), WhatsApp (28.3%), Instagram (25.8%) and Twitter (20.7%). The least used medium was the radio (9%) ^(12)^.

The urgent and pervasive spread of information about the COVID-19 pandemic was associated with feelings of depression and fear^(11,13,16)^. The results obtained in this study reinforce scientific evidence, particularly concerning older adults. Older adults who were frequently exposed to news and information about COVID-19 on television and social media and used these sources for two to six hours or more were more commonly associated with depression.

Researchers in a study with 1,577 community-dwelling adults and 214 healthcare providers investigated risk factors for depression in Wuhan, China, the epicenter of the pandemic. This study found that exposure to news about COVID-19 on social media for two or more hours daily was associated with depression in adults (37.16% n=586). The study cautions that social media can play a specific role in mental distress during epidemics^(16)^. Another study in Singapore with 1,145 adults showed that depression scores were positively related to increased time spent receiving updates on COVID-19 on social media and television^(13)^.

A scoping review analyzed 33 articles on infodemics and mental health in adults and older adults, noting the signs and symptoms most frequently mentioned in the analyzed publications. It was observed that depression appeared in 51.5% of the publications. This result reinforces the idea that excessive exposure to information is directly related to the development of mental health issues^(11)^.

Despite the trend in the scientific literature to attribute depression symptoms to the use of Information and Communication Technologies (ICTs), it is important to consider reverse causality. In this case, it is believed that depression may lead to online browsing for information as a way to pass the time or a form of distraction^(16,28-29)^. A study conducted in Canada and the United States with 685 adults found that the use of social media and television was higher among the 621 respondents who reported feeling fearful, worried or experiencing other symptoms of mental distress. Respondents who rated their mental health as “poor” were twice as likely to use computers and cell phones as coping tools during self-isolation^(28)^.

The user’s need for information may be linked to the “cognitive void.” Perception and interpretation of this “void,” characterized by uncertainty and the need to seek information, shapes the individual’s behavior in accessing it. In other words, there is a desire to fill the existing gap and, in doing so, attempt to overcome this “absence”^(30)^.

A review on the impact of social media on perceived loneliness and/or social isolation experienced by older adults concluded that the scientific evidence suggests social media use may reduce feelings both aspects. It can provide greater contact between older adults and their families, serve as a source of support, contribute to a greater sense of control over their lives and foster a stronger sense of community belonging. The study stresses that the internet may play an important role in reducing isolation barriers^(31)^. However, in an infodemic context, the constant search to fill gaps with conclusive information about problems may end up generating misinformation and disinformation.

Contemporary studies on the “performance society” emphasize that it is largely based on information consumption. In the performance society, sustained by digital culture, the prevailing principle is: “where there’s a will, there’s a way”. It suggests that, nowadays, as long as you want to, you can have access to news about any worldwide event. For older adults, understanding this new information model may cause distress about being and playing the social role of information consumers and communication mediators. Consequently, they are, even if unintentionally, included in this framework^(32-32)^.

Nonetheless, this study showed that older adults who reported not being exposed to information on social media and television also showed signs of depression. Avoiding information may give people a sense that they are missing out on important new information, which may generate stress and anxiety, thus becoming a maladaptive coping strategy^(33)^.

A study conducted with 1,059 Germans who were asked to report their feelings and behaviors since the beginning of the COVID-19 pandemic revealed that the choice to avoid information may be linked to the feeling that nothing that can be done to prevent the negative consequences of the disease. Participants who were “very concerned” about the risks to mental and physical health were also more likely to avoid or ignore the media. The analysis also showed that higher levels of stress, distress and depression were associated with less access to social media^(34)^.

A limitation of the study relates to the use of a web-based survey for data collection, which, consequently, negatively affected participation from a significant number of older adults who do not have access to the internet and social media, leading to sampling bias.

The study provides contributions and advancements for practice and research and their political implications. Regarding practice, the results highlight the need for nursing interventions to address the consequences of the infodemic on the mental health of older adults. They also emphasize the need to develop health education strategies tailored for older adults related to the infodemic, through the clear and objective disclosure of accurate and necessary information, aiming for a better quality of life.

The research highlights the importance of promoting and implementing tools for managing infodemics, aimed at monitoring their impacts on the communication of health emergencies. Managing information overload should be a topic of discussion in public health, as the infodemic phenomenon is complex, situated in a multifactorial context influenced by political and sociocultural issues.

## Conclusion

It was concluded that in terms of demographic profile, the investigated sample consisted mostly of older women, aged 60 to 64, white, highly educated, living with one or two people in their own homes, receiving a pension and with no change in income due to the pandemic. An association was found between exposure to news and information about COVID-19 and the presence of depression symptoms in older women frequently exposed to COVID-19-related information on television and social media for periods ranging from two to six hours. The infodemic in a context of pandemic and health emergency may have a negative impact on the mental health of older adults.

This study contributes to addressing research gaps regarding the relationship between infodemics and depression screening in older adults. The identified mediators can be targeted through psychological interventions to prevent the deterioration of mental health in older adults exposed to information overload. Additionally, the findings support the development of coping strategies and enhanced research focused on understanding and analyzing the cognitive and behavioral aspects of information consumption among older adults, integrating these insights into psychosocial care practices.
