# Supplementary material for: Association between the COVID-19 infodemic and depression symptom screening in older adults
Source: Rev Lat Am Enfermagem. 2025 May 19;33:e4461. doi: 10.1590/1518-8345.7454.4461 (PMC12088656; doi:10.1590/1518-8345.7454.4461)
Supplement: Translation

# Translation: Associação entre infodemia de COVID-19 e o rastreio para sintomas depressivos em pessoas idosas*

**DOI:** 10.1590/1518-8345.7454.4462

**Authors:** Patricia Rodrigues Braz, Tiago Ricardo Moreira, Andréia Queiroz Ribeiro, Rosimere Ferreira Santana, Jack Roberto Silva Fhon, Alexandre Favero Bulgarelli, Vilanice Alves de Araújo Püschel, Edna Aparecida Barbosa de Castro, Denise Rocha Raimundo Leone, Ricardo Bezerra Cavalcante

**Affiliations:** 1 Universidade Federal de Juiz de Fora, Juiz de Fora, MG, Brasil; 2 Universidade Federal de Viçosa, Viçosa, MG, Brasil; 3 Universidade Federal Fluminense, Niterói, RJ, Brasil; 4 Universidade de São Paulo, Escola de Enfermagem, São Paulo, SP, Brasil; 5 Universidade Federal do Rio Grande do Sul, Porto Alegre, RS, Brasil

**Keywords:** Infodemia, COVID-19, Saúde do idoso, Saúde mental, Depressão, Transtorno depressivo

## Abstract

analisar o perfil de exposição às informações sobre a COVID-19 e sua associação com o rastreio para sintomas depressivos em uma amostra de pessoas idosas no Brasil.

estudo transversal com dados coletados por *web-based survey*, com 3.307 participantes recrutados via redes sociais e *e-mail*. Realizou-se análise descritiva e bivariada para estimar associações de interesse e regressão logística bruta e ajustada por variáveis previsoras, sociodemográficas e infodêmicas.

foi encontrada associação significativa entre o desfecho para rastreio de sintomas depressivos entre as pessoas idosas que se expuseram por três a seis horas ou mais a redes sociais e televisão e entre as que afirmaram não se expor a notícias e informações sobre COVID-19 divulgadas via televisão.

as mulheres idosas frequentemente expostas às informações relacionadas à COVID-19 pela televisão e redes sociais, por períodos entre duas e seis horas, apresentaram sintomas depressivos. Este estudo traz contribuições para a pesquisa sobre infodemia e saúde mental, uma vez que contempla um eixo lacunar de investigação sobre a relação entre rastreio de sintomas depressivos em uma amostra de pessoas idosas e sua associação com o perfil de exposição às informações sobre COVID-19.

## Destaques

**(1)** Existe associação entre as variáveis infodêmicas e o rastreio para depressão. **(2)** Ineditismo na investigação sobre os impactos da infodemia no rastreio para depressão. **(3)** Os idosos que não se expuseram às notícias também foram rastreados para depressão. **(4)** O estudo apresenta contribuições para a pesquisa sobre infodemia e saúde mental.

## Introdução

A COVID-19 foi considerada um novo tipo de afecção viral. No período de sua eclosão, o cenário era incipiente sobre informações científicas e advindas de instituições governamentais sobre a doença; nesse contexto, a disseminação excessiva de informações foi potencializada pelas mídias. Esse processo foi caracterizado como uma infodemia, termo que se refere ao excesso de informações associadas a um assunto específico, que podem se multiplicar exponencialmente em pouco tempo devido a um evento específico^(1-2)^, e que propicia, ainda, o surgimento de informações erradas e desinformações, além da manipulação de informações com intenção duvidosa^(2)^. A infodemia de COVID-19 foi amplamente reconhecida por ter agravado o contexto da pandemia, configurando-se como um fenômeno potencializador da propagação de conteúdos inidôneos^(3)^.

Pessoas idosas, especialmente no Brasil, podem ser mais vulneráveis à desinformação e, consequentemente, contribuir para a difusão de notícias falsas. Não necessariamente pela idade, mas em razão de algumas características que tendem a ser mais comuns nessa etapa do curso de vida.

Há um conjunto de características que parecem tornar as pessoas idosas do Brasil mais vulneráveis ao consumo e à disseminação de *fake news* sobre COVID-19. O analfabetismo funcional é um fator interveniente. No Brasil, a última pesquisa realizada sobre analfabetismo funcional, em 2018, apresentou que 10,3% do grupo populacional com a faixa etária de 60 anos ou mais se enquadrava como analfabetos funcionais^(4)^. Além disso, há fragilidades no letramento em saúde e no letramento digital, somadas à baixa alfabetização midiática e à mínima alfabetização científica^(5-7)^.

Essa conjuntura de infodemia tem acarretado repercussões negativas para a saúde mental da população idosa. Estudos^(8-13)^ realizados com amostras de adultos identificaram o crescimento de casos de depressão associados ao excesso informacional, bem como recidivas de casos de depressão causada por informações negativas sobre a COVID-19^(13-14)^. Adicionalmente, há evidências de que indivíduos deprimidos tendem a usar frequentemente as redes sociais^(15)^.

Durante a pandemia, o diagnóstico de depressão elevou-se mundialmente^(16-18)^. No primeiro ano desta, a prevalência global de depressão aumentou 25%. Antes da pandemia, no início de 2020, cerca de 193 milhões pessoas tiveram transtorno depressivo maior, mas após o advento dela, estimativas mostraram um salto para 246 milhões para transtorno depressivo maior, representando um aumento de 28% no ano de 2021^(17)^. Atualmente, na América Latina, o Brasil é o país com maior prevalência de depressão e o segundo nas Américas^(18)^, tendo a maior prevalência da doença na população idosa^(19)^.

Os critérios diagnósticos de episódio depressivo pela Classificação Internacional de Doenças (CID-10) contemplam rebaixamento do humor, redução da energia, diminuição da atividade, alteração da capacidade de experimentar o prazer, perda de interesse, diminuição da capacidade de concentração, associadas à fadiga. No episódio depressivo leve, costumam-se estar presentes ao menos dois ou três desses sintomas, já no episódio depressivo grave sem sintomas psicóticos, vários dos sintomas são marcantes e angustiantes, envolvendo a perda da autoestima e o sentimento de culpa. Ademais, ideações suicidas são comuns e observa-se, em geral, uma série de sintomas somáticos^(20)^.

Ante o exposto, os estudos demonstram que a infodemia de COVID-19 impactou negativamente na saúde mental das pessoas idosas, logo é relevante investigar tal fenômeno e suas repercussões na saúde mental dessa população acerca do rastreio de sintomas depressivos; outro ponto é que estudos nesse eixo e que trazem pesquisas com a população idosa ainda são escassos. A pesquisa aqui proposta teve como objetivo analisar o perfil de exposição às informações sobre COVID-19 e sua associação com o rastreio para sintomas depressivos em uma amostra de pessoas idosas no Brasil.

## Método

### Cenário e período

Trata-se de um estudo descritivo, exploratório e transversal, realizado com pessoas idosas no Brasil. A coleta de dados ocorreu entre julho de 2020 e março de 2021.

### Amostra e critérios de seleção

O tamanho amostral do estudo multicêntrico foi estimado por município, considerando a população local de pessoas idosas, a partir da fórmula: n = N. Z².p.(1-p) /Z².p.(1- p). + e². (N-1), em que “n” é o tamanho amostral calculado, “N” é o tamanho da população, “Z” a variável normal padronizada associada ao nível de confiança (nesse caso, 95%), “p” a verdadeira probabilidade do evento (P = (1-P) = 0.5, suposição de probabilidade máxima) e “e” o erro amostral, sendo utilizado erro amostral de 5%. A amostra para dados parciais considerou: 50% de prevalência do fenômeno, 3% de erro, 95% de nível de confiança + 10% de análises múltiplas e 20% considerando possíveis perdas dentro da amostra (respostas em branco). A amostragem foi não probabilística no Brasil. Inicialmente, o banco de dados contou com 3321 participantes, sendo excluídas duplicações de informações e participantes que não preencheram todas as informações, obtendo-se um total de 3.307 pessoas idosas brasileiras participantes.

Os participantes elegíveis para este estudo foram pessoas idosas brasileiras que tinham 60 anos ou mais, acesso a redes sociais, e-mail e/ou telefone e habilidade para responder ao questionário via redes sociais ou pelo telefone. O critério de exclusão foi declarar não possuir habilidades para responder ao questionário pelas mídias digitais ou mesmo pelo telefone, bem como pessoas idosas residentes em Instituições de Longa Permanência para Idosos (ILPIs), compreendendo-se o fato de que o processo de institucionalização do idoso por si só pode contribuir para o desenvolvimento ou agravamento de quadros depressivos e ansiosos^(21)^.

Foram utilizadas diferentes estratégias para garantir maior representatividade da amostra, valendo-se da estratégia de bola de neve virtual, por meio do compartilhamento do link para participação da pesquisa com sociedades científicas de geriatria e gerontologia, unidades de atenção à saúde, associações de aposentados e, de maneira direta, as pessoas idosas que já eram acompanhadas por meio de atividades de pesquisa e extensão nos centros colaboradores da pesquisa.

### Instrumentos utilizados para a coleta das informações e variáveis do estudo

O instrumento de coleta de dados sobre exposição às informações sobre COVID-19 foi adaptado de estudos prévios sobre infodemia^(8,22)^. Além de variáveis socioeconômicas, havia as variáveis relacionadas à infodemia sobre COVID-19 nas redes sociais, no rádio e na televisão, que se referiam ao tipo de mídia acessada e tempo de exposição às mídias (frequência e horas).

Para avaliar o rastreio de sintomas depressivos, utilizou-se a Escala de Depressão Geriátrica (EDG). Foi adotado o ponto de corte 5/6 (não caso/caso), que é apontado como adequado para rastreamento, identificando precocemente e categorizando sintomas depressivos em pessoas idosas em ambientes não especializados no Brasil^(23)^.

### Coleta de dados

O instrumento de coleta de dados foi estruturado por meio de um *web-based survey*. No primeiro acesso ao *link*, as pessoas idosas eram direcionadas ao Termo de Consentimento Livre e Esclarecido (TCLE) digital para que pudessem ler e optar por aceitar ou recusar a participação no estudo. A opção de participar, ou não, foi registrada automaticamente no banco de dados gerado pela *web-based survey.* Os que optavam por seguir com a participação tinham acesso às perguntas da pesquisa.

### Tratamento e análise dos dados

Os procedimentos de tratamento e análise foram realizados pelo programa *Statistical Package for the Social Sciences* (SPSS), versão 23.0. As análises descritivas foram feitas mediante caracterização demográfica dos participantes e das variáveis referentes à exposição a notícias e informações relacionadas à COVID-19 em meios de comunicação. Para as variáveis qualitativas, foram estimadas as frequências absolutas e relativas. Para as variáveis quantitativas, foram computadas medidas de posição (média e mediana) e de dispersão (desvio-padrão, intervalo interquartílico, mínimo e máximo), conforme a distribuição dos dados (simétrica ou assimétrica).

A associação entre variáveis infodêmicas e os desfechos relacionados ao rastreamento para sintomas depressivos foi avaliada por meio do teste qui-quadrado. Posteriormente, realizou-se a análise de regressão logística múltipla bruta e ajustada (por sexo, faixa etária, escolaridade, coabitação e alterações de renda na pandemia) para a associação entre as variáveis independentes de interesse e o desfecho para depressão, com respectivos intervalos de confiança de 95%. O nível de significância adotado em todos os testes foi de 5%.

### Aspectos éticos

O estudo foi aprovado pela Comissão Nacional de Ética em Pesquisa (Conep) do Brasil em 03/07/2020 – Certificado de Apresentação de Apreciação Ética (CAAE): 31932620.1.1001.5147, sob o Parecer nº 4.134.050 e, após a aprovação, deu-se início à coleta de dados. Está previsto o atendimento de todos os requisitos éticos e legais próprios de investigações envolvendo seres humanos, em consonância com as disposições regulamentadoras da Resolução n.º 466/12 brasileira do Conselho Nacional de Saúde.

Aos entrevistados foi garantido o anonimato, por meio da utilização numérica para representá-los no estudo, a elucidação sobre o objetivo, justificativa e procedimentos da pesquisa, bem como a explicação sobre a participação voluntária, sem vantagens financeiras ou gastos, e a informação sobre a divulgação dos achados, que ocorrerá apenas em eventos e/ou periódicos de natureza científica.

## Resultados

Participaram do estudo 3307 pessoas idosas, sendo 68,4% do sexo feminino, 38,9% pertencentes à faixa etária de 60 a 64 anos, 55,5% casados e 71,5% autodeclarados brancos. No que se refere à moradia, 95,6% residiam em área urbana e 57,0% coabitavam com uma ou duas pessoas em seus domicílios. Dos participantes, apenas 8,9% não estudaram ou não concluíram o ensino básico, enquanto 19,5% tinham ensino superior completo. A maioria (40,6%) utilizava serviços de saúde tanto gratuitos quanto pagos, e 73,8% dos participantes responderam que a pandemia não afetou sua renda mensal.

Acerca do rastreio para sintomas depressivos relacionado às variáveis sociodemográficas, destaca-se que 66,7% das pessoas idosas que preferiram não declarar o sexo foram rastreadas com sintomas depressivos, enquanto 44,7% das pessoas idosas mais longevas, com 80 anos ou mais, apresentaram rastreio para sintomas depressivos, sendo esse o subgrupo etário com maior percentual. No que se refere ao estado civil, 46,3% das pessoas idosas solteiras apresentaram rastreio para sintomas depressivos. Em relação à coabitação, 37,3% das pessoas idosas que moravam sozinhas foram rastreadas com sintomas depressivos e acerca da escolaridade e anos de estudo, 47,1% das pessoas idosas que não tiveram oportunidade de estudar ou concluir o ensino básico foram rastreadas com sintomas depressivos (Tabela 1).

**Tabela 1 -:** Perfil demográfico e de rastreio para sintomas depressivos nas pessoas idosas participantes da pesquisa (n = 3307). Brasil, 2021

**Variáveis**	**N**	**%**	**Rastreamento para sintomas depressivos**			
			**Não-caso**		**Caso**	
			**N**	**%**	**N**	**%**
**Sexo**						
Feminino	2.250	68,4	1347	59,9%	903	40,1%
Masculino	1.039	31,6	638	61,4%	401	38,6%
Prefiro não declarar	18	0,5	6	33,3%	12	66,7%
**Faixa etária (anos)**						
60 a 64	1.285	38,9	799	62,2%	486	37,8%
65 a 69	921	27,9	543	59,0%	378	41,0%
70 a 74	503	15,2	309	61,4%	194	38,6%
75 a 79	334	10,1	194	58,1%	140	41,9%
80 ou mais	264	8,0	146	55,3%	118	44,7%
**Estado civil**						
Casado(a)/morando junto	1.835	55,5	1119	61,0%	716	39,0%
Viúvo(a)	598	18,1	340	56,9%	258	43,1%
Separado(a)/desquitado(a)	509	15,4	336	66,0%	173	34,0%
Solteiro (a)	365	11,0	196	53,7%	169	46,3%
**Raça/cor**						
Branco	2,364	71,5	1436	60,7%	928	39,3%
Não branco	943	28,5	555	58,9%	388	41,1%
**Coabitação**						
Mora sozinho (a)	587	17,8	368	62,7%	219	37,3%
Uma ou duas pessoas	1.886	57,0	1129	59,9%	757	40,1%
Três ou mais pessoas	834	25,2	494	59,2%	340	40,8%
**Residência própria**						
Sim	2.756	83,3	1668	60,5%	1088	39,5%
Não	551	16,7	323	58,6%	228	41,4%
**Área de residência**						
Zona urbana	3.160	95,6	1910	60,4%	1250	39,6%
Zona rural	147	4,4	81	55,1%	66	44,9%
**Escolaridade máxima**						
Não estudou ou não concluiu o ensino básico	295	8,9	156	52,9%	139	47,1%
Ensino básico ou fundamental	713	21,6	409	57,4%	304	42,6%
Ensino médio	718	21,7	449	62,5%	269	37,5%
Ensino superior completo	645	19,5	389	60,3%	256	39,7%
Especialização	512	15,5	322	62,9%	190	37,1%
Mestrado e/ou doutorado	424	12,8	266	62,7%	158	37,3%
**Serviços de saúde utilizados**						
Apenas serviços pagos de saúde (incluindo plano de saúde)	1.133	34,3	692	61,1%	441	38,9%
Ambos (gratuitos e pagos)	1.343	40,6	823	61,3%	520	38,7%
Apenas serviços gratuitos de saúde	814	24,6	466	57,2%	348	42,8%
Nenhum	17	0,5	10	58,8%	7	41,2%
**Recebe aposentadoria ou pensão**						
Sim	2.565	77,6	1520	59,3%	1045	40,7%
Não	740	22,40	470	63,5%	270	36,5%
**Pandemia alterou a renda**						
Não	2.437	73,8	1469	60,3%	968	39,7%
Sim, a minha renda diminuiu	787	23,8	47	58,8%	33	41,2%
Sim, a minha renda aumentou	80	2,4	474	60,2%	313	39,8%

Sobre os recursos mais utilizados para acessar notícias e informações sobre a COVID-19 durante o dia: 81,1% das pessoas idosas utilizaram a televisão, 58,8% as redes sociais e apenas 26,48% utilizaram o rádio. As pessoas idosas também reportaram terem percebido a exposição às informações e notícias sobre COVID-19 no período de uma semana (sete dias), a partir de respostas como “nenhuma exposição” até “exposição frequente”. Foi reportada como fonte “frequente” de exposição à televisão (44,5%), seguida por “algumas vezes” das redes sociais (44,3%). Contrariamente, o rádio não representou fonte de exposição para a maioria das pessoas idosas (59,1%). Sobre as horas de exposição, 39,3% das pessoas idosas se expuseram por três horas ou mais à televisão, enquanto 32,8% acessaram as redes sociais por duas a cinco horas e 37,0% se expuseram por uma hora ou mais ao rádio (Tabela 2).

Foi encontrada associação significativa (*P* > 0,05, ou seja, probabilidade maior que 5%) entre o desfecho rastreio para presença de sintomas depressivos e as variáveis frequência de exposição à televisão (*P* = 0,01), horas de exposição a notícias e informações sobre COVID-19 nas redes sociais (*P* =0,00 ) e na televisão (*P* =0,02). No que concerne à variável frequência de exposição à televisão, 42,9% das pessoas idosas que relataram não se expor às informações foram rastreadas para presença de sintomas depressivos. Já sobre as horas de exposição, 44,3% das pessoas idosas que referiram se expor por seis horas ou mais a notícias e informações sobre COVID-19 nas redes sociais foram rastreadas para presença de sintomas depressivos. E 43,2% que referiram se expor por três horas ou mais a notícias e informações sobre COVID-19 na televisão também apresentaram rastreio para presença de sintomas depressivos (Tabela 3).

**Tabela 2 -:** Caracterização dos meios de informação mais utilizados para acessar notícias e informações sobre COVID-19 pelas pessoas idosas participantes (n = 3307). Brasil, 2021

**Meios de informação**	**N**	**%**
**Redes sociais (N=3.303)**		
Sim	1.943	58,8
Não	1.361	41,2
**Frequência de exposição**		
Nenhuma	822	24,9
Algumas vezes	1.464	44,3
Frequentemente	1.021	30,9
**Horas de exposição a notícias e informações sobre COVID-19 nas redes sociais**		
Nenhuma	848	25,6
Uma	811	24,5
Duas a cinco	1084	32,8
Seis ou mais	560	16,9
**Televisão (N=3.304)**		
Sim	2.680	81,1
Não	624	18,9
**Frequência de exposição**		
Nenhuma	394	11,9
Algumas vezes	1.440	43,5
Frequentemente	1.473	44,5
**Horas de exposição a notícias e informações sobre COVID-19 na televisão**		
Nenhuma	431	13,0
Uma	884	26,7
Duas	685	20,7
Três ou mais	1301	39,3
**Rádio (N=3.304)**		
Sim	876	26,5
Não	2.429	73,5
**Frequência de exposição**		
Nenhuma	1.956	59,1
Algumas vezes	956	28,9
Frequentemente	395	11,9
**Horas de exposição a notícias e informações sobre COVID-19 no rádio**		
Nenhuma	2083	63,0
Uma ou mais	1223	37,0

**Tabela 3 -:** Associação entre variáveis infodêmicas e o rastreamento para presença de sintomas depressivos entre as pessoas idosas participantes (n = 3307). Brasil, 2021

**Variável**	**Presença de sintomas depressivos**				**Valor de *P***
	**Não caso**		**Caso**		
	**N**	**%**	**N**	**%**	
**Frequência de exposição a redes sociais**					0,08
Nenhuma	463	56,3%	359	43,7%	
Algumas vezes	920	62,8%	544	37,2%	
Frequentemente	608	59,5%	413	40,5%	
**Frequência de exposição à televisão**					0,01
Nenhuma	225	57,1%	169	42,9%	
Algumas vezes	909	63,1%	531	36,9%	
Frequentemente	857	58,2%	616	41,8%	
**Frequência de exposição a rádio**					0,07
Nenhuma	1182	60,4%	774	39,6%	
Algumas vezes	591	61,8%	365	38,2%	
Frequentemente	218	55,2%	177	44,8%	
**Horas de exposição a notícias e informações sobre COVID-19 nas redes sociais**					0,00
Nenhuma	482	56,8%	366	43,2%	
Uma	529	65,2%	282	34,8%	
Duas a cinco	665	61,3%	419	38,7%	
Seis ou mais	312	55,7%	248	44,3%	
**Horas de exposição a notícias e informações sobre COVID-19 na televisão**					0,02
Nenhuma	254	58,9%	177	41,1%	
Uma	573	64,8%	311	35,2%	
Duas	421	61,5%	264	38,5%	
Três ou mais	739	56,8%	562	43,2%	
**Horas de exposição a notícias e informações sobre COVID-19 no rádio**					0,83
Nenhuma	1251	60,1%	832	39,9%	
Uma ou mais	739	60,4%	484	39,6%	

A análise demonstrou que, na regressão logística bruta, foram associados ao rastreamento para presença de sintomas depressivos estar exposto frequentemente às notícias e informações sobre COVID-19 pelo rádio (*P* = 0,02) e televisão (*P* = 0,00), bem como estar exposto por duas horas (*P* =0,00) ou por três horas ou mais (*P* = 0,04) à televisão e estar exposto por duas a cinco horas (*P* = 0,00) ou por seis horas ou mais (*P* = 0,02) às redes sociais. Já na análise ajustada, as variáveis infodêmicas que permaneceram associadas ao rastreio para presença de sintomas depressivos (*P* ≤ 0,05) foram: a exposição frequente à televisão e ao rádio; a exposição por duas a cinco horas ou por seis horas ou mais às redes sociais e exposição por duas horas à televisão (Tabela 4).

**Tabela 4 -:** Modelo de Regressão Logística bruta e ajustada para o somatório da Escala de Depressão Geriátrica aplicada às pessoas idosas participantes (n = 3307). Brasil, 2021

**Presença de sintomas depressivos (regressão logística)**		
	Análise Bruta OR(IC95%)*	Análise Ajustada OR(IC95%)*
**Frequência de exposição a redes sociais**		
Nenhuma	1	1
Algumas vezes	1,14 (0,94 - 1,37)	1,01 (0,82- 1,25)
Frequentemente	0,87 (0,73 - 1,02)	0,85 (0,72- 1,01)
**Frequência de exposição à televisão**		
Nenhuma	1	1
Algumas vezes	1,04 (0,83- 1,30)	1,06 (0,85- 1,34)
Frequentemente	0,81 (0,70 -0,94)	0,81 (0,70- 0,94)
**Frequência de exposição a rádio**		
Nenhuma	1	1
Algumas vezes	0,80 (0,64 - 1,00)	0,80 (0,64- 1,00)
Frequentemente	0,76 (0,60 – 0,96)	0,74 (0,58- 0,95)
**Horas de exposição a notícias e informações sobre COVID-19 nas redes sociais**		
Nenhuma	1	1
Uma	0,95 (0,77 -1,18)	0,86 (0,68- 1,09)
Duas a cinco	0,67 (0,53 -0,83)	0,68 (0,54 - 0,85)
Seis ou mais	0,79 (0,64 - 0,97)	0,80 (0,64- 0,98)
**Horas de exposição a notícias e informações sobre COVID-19 na televisão**		
Nenhuma	1	1
Uma	0,91 (0,73- 1,14)	0,95 (0,76 - 1,20)
Duas	0,71 (0,59 -0,85)	0,73 (0,61 - 0,87)
Três ou mais	0,82 (0,68 - 0,99)	0,85 (0,70 - 1,02)
**Horas de exposição a notícias e informações sobre COVID-19 no rádio**		
Nenhuma	1	1
Uma ou mais	1,01 (0,87 -1,17)	1,03 (0,89 - 1,20)

*OR(IC95%) = *Odds ratio* e intervalo de confiança

## Discussão

O estudo almejou investigar a infodemia de COVID-19 e suas repercussões na saúde mental das pessoas idosas acerca do rastreio para presença de sintomas depressivos.

No que se refere às variáveis infodêmicas, a reflexão sobre o cenário temporal da pandemia se faz relevante. No período de desenvolvimento do estudo, entre julho de 2020 a março de 2021, o cenário epidemiológico da COVID-19 era alarmante. No que concerne aos dados epidemiológicos, segundo o Ministério da Saúde^(24)^ brasileiro, a média de óbitos era de aproximadamente 1.026 no segundo semestre de 2020; o documento mostra ainda que o mês de março de 2021 concentrou o maior percentual de óbitos, sendo a média de 2.207 óbitos por dia^(24)^.

As regras de distanciamento social ainda estavam em vigência, o que fomentou o maior tempo de consumo midiático e o aumento no hábito de assistir à televisão entre a população adulta. As informações sobre o número de óbitos e infectados estavam em destaque na mídia, assim como, nesse contexto, havia também informações relacionadas à disputa política sobre a compra e distribuição das vacinas, visto que os primeiros imunizantes só chegaram ao país em janeiro de 2021^(8,12)^.

Os resultados desta pesquisa identificaram que a televisão foi o meio mais utilizado para o acesso às informações e também foi referida como a fonte de informação de maior frequência de exposição pelas pessoas idosas, sendo que a exposição perdurava por três horas ou mais, seguida das redes sociais. Já o rádio foi a fonte de informação menos acessada.

Um estudo realizado no Brasil durante a pandemia da COVID-19, composto por 45.161 indivíduos, dos quais 20,3% eram pessoas idosas com 60 anos ou mais, verificou aumento no uso de televisão, sendo as pessoas idosas o grupo com o maior tempo médio diante das telas televisivas. A pesquisa aduz ainda que 61,6% dos participantes mantiveram-se expostos por três horas ou mais a esse meio^(25)^.

Outra investigação analisou o comportamento de busca por informação por pessoas idosas durante a pandemia de COVID-19 e identificou que a televisão foi o principal veículo de comunicação referido por elas^(26)^. As pessoas idosas entrevistadas enunciaram que a predileção pela televisão se dava pela facilidade de acesso, bem como pela rapidez com que obtinham informações, em razão das atualizações diante dos acontecimentos diários em todo o mundo^(27)^.

No caso das pessoas idosas, a televisão continua sendo o meio mais simples, rápido e de fácil acesso a informações^(3,12,26)^, visto que idosos podem ter maior dificuldade para usar e manipular computadores e celulares.

Outra investigação feita com 799 participantes adultos no Rio Grande do Sul também constatou que o meio mais utilizado pelos participantes para acessar informações sobre a COVID-19 foi a televisão (76,3%), seguida das redes sociais, mais precisamente pelo uso do *Facebook* (37%), *WhatsApp* (28,3%), *Instagram* (25,8%), *Twitter* (20,7%), entre outros. Novamente, o meio menos utilizado foi o rádio (9%)^(12)^.

A urgência de informações e a difusão informacional generalizada sobre a pandemia da COVID-19 se associaram a sentimentos de depressão e medo^(11,13,16)^. Os resultados obtidos neste estudo ratificam as evidências científicas, notadamente no que concerne às pessoas idosas. Pessoas idosas que estavam expostas frequentemente às notícias e informações sobre COVID-19 fornecidas pela televisão e redes sociais e que utilizavam esses meios durante duas a seis horas ou mais foram comumente rastreadas para depressão.

Os pesquisadores de um estudo realizado com 1.577 adultos comunitários e 214 profissionais de saúde investigaram os fatores de risco para depressão em pessoas da comunidade e entre profissionais de saúde no epicentro Wuhan, na China. Evidenciou-se que ficar exposto por duas horas diárias ou mais a notícias sobre a COVID-19 via mídia social se associou a esse transtorno em adultos (37,16% n = 586). O estudo alerta que mídias sociais podem ter um papel específico no sofrimento mental da população durante epidemias^(16)^. Outra pesquisa feita em Cingapura, com 1.145, adultos demonstrou que os escores de depressão se relacionaram positivamente com o aumento do tempo gasto recebendo atualizações sobre a COVID-19 nas redes sociais e televisão^(13)^.

Uma revisão de escopo analisou 33 artigos sobre infodemia e saúde mental de adultos e pessoas idosas, enfatizando os sinais e sintomas mais enunciados nas publicações analisadas. Observou-se que a depressão aparece em 51,5% das publicações. Esse resultado reforça a ideia de que a exposição excessiva a informações está diretamente relacionada ao desenvolvimento de problemas de saúde mental^(11)^.

Ainda que exista na literatura científica uma tendência de atribuir ao uso de Tecnologias da Informação e Comunicação (TICs) os sintomas de depressão, é preciso considerar a causalidade reversa. Nesse caso, considera-se que a depressão pode levar à ruminação nas mídias sociais em busca de informações, seja para ocupação do tempo ocioso ou para distração^(16,28-29)^. Uma pesquisa realizada no Canadá e nos Estados Unidos, com 685 adultos, verificou que o uso de redes sociais e televisão foi maior entre os 621 entrevistados que declararam que estavam com medo, preocupados e apresentando outros sintomas de sofrimento mental. Os entrevistados que consideraram sua saúde mental “ruim” tiveram duas vezes mais chances de optar pelo uso de computadores e celulares como ferramentas de enfrentamento do autoisolamento^(28)^.

A necessidade informacional do usuário pode estar ligada ao “vazio cognitivo”. A percepção e interpretação desse “vazio”, caracterizado pela incerteza e necessidade de busca, modula o comportamento do indivíduo ao acesso de informações, ou seja, há o desejo de completar a lacuna existente e, assim, tentar transpor essa “ausência”^(30)^.

Uma revisão sobre o impacto de mídias sociais na percepção de solidão e/ou no isolamento social vivido por idosos concluiu que as evidências científicas demonstraram que o uso de mídias sociais digitais pode reduzir esses dois pontos e favorecer maior contato entre idosos e famílias, servindo como fonte de apoio e contribuindo para maior senso de controle de suas vidas e para maior senso de pertencimento comunitário. O estudo enfatiza que a internet pode ser considerada importante na diminuição das barreiras de isolamento^(31)^. Porém, em um cenário infodêmico, a busca constante por preencher lacunas conclusivas sobre problemas pode acabar gerando a desinformação e a má informação.

Nos estudos contemporâneos sobre a “sociedade do desempenho”, ressalta-se que essa sociedade se estrutura em grande medida pelo consumo de informação, sendo mantida na cultura digital a retórica do querer é poder se sobrepujar. Essa premissa suscita a ideia de que, atualmente, só não tem acesso à informação sobre um acontecimento no mundo quem não quer. Para as pessoas idosas, a compreensão desse novo modelo informacional pode causar conflitos internos sobre ser e estar no lugar social como consumidores de informação e mediadores de comunicação. Por conseguinte, eles são, mesmo que não intencionalmente, contemplados nessa estrutura^(32-33)^.

Não obstante, esta pesquisa demonstrou que as pessoas idosas que relataram não se expor às informações nas redes sociais e televisão também foram rastreadas para depressão. A evasão das informações pode levar as pessoas à percepção de que elas estão perdendo novas informações importantes, fato esse que pode gerar estresse e ansiedade, sendo uma atitude de enfrentamento mal adaptativa^(33)^.

Uma pesquisa realizada com 1.059 alemães que foram instruídos a relatar suas emoções e comportamento desde o início da pandemia COVID-19 resultou na compreensão de que a opção por evitar informações pode estar ligada ao sentimento de que não há nada que se possa fazer para prevenir consequências negativas da doença. Os participantes que estavam “muito preocupados” com os riscos à saúde mental e física também estavam mais propensos a evitar ou ignorar a mídia. A análise revelou, ainda, que maior nível de estresse, ansiedade e depressão foi associado ao menor acesso às redes sociais^(34)^.

Uma limitação do estudo refere-se ao uso da *web-based survey* na coleta de dados, que, consequentemente, interferiu negativamente na participação da pesquisa de uma parcela significativa de pessoas idosas que não têm acesso à internet e às redes sociais, o que culminou em um viés amostral.

O estudo traz contribuições e avanços para os campos da prática, pesquisa e seus desdobramentos políticos. Na prática, os resultados evidenciam a necessidade de intervenções de Enfermagem frente às repercussões da infodemia na saúde mental das pessoas idosas e ensejam a necessidade de desenvolver estratégias adequadas às pessoas idosas de educação em saúde no que se refere à infodemia, por meio da difusão de forma clara e objetiva de informações idôneas e necessárias, visando melhor qualidade de vida.

Já na pesquisa, os resultados ressaltam a importância de promover e aplicar ferramentas para a gestão de infodemias, com o intuito de monitorar seus impactos nos cenários de emergências em saúde no âmbito da comunicação. O gerenciamento do excesso informacional deve ser uma pauta em debate na saúde pública, visto que o fenômeno infodêmico apresenta complexidade por estar centralizado em um contexto multifatorial, atravessado por questões políticas e socioculturais influentes.

## Conclusão

Concluiu-se que o perfil demográfico da amostra investigada foi, em sua maioria, mulheres idosas, de 60 a 64 anos, brancas, de alta escolaridade, coabitando com uma ou duas pessoas em residência própria, recebendo aposentadoria e sem alteração na renda pela pandemia. Verificou-se também a associação entre a exposição a notícias e informações sobre COVID-19 e a presença de sintomas depressivos nas mulheres idosas frequentemente expostas a essas informações relacionadas à COVID-19 pela televisão e pelas redes sociais, por períodos de duas a seis horas. A infodemia em contexto pandêmico frente a um cenário de emergências em saúde pode repercutir negativamente na saúde mental de pessoas idosas.

Este estudo apresenta contribuições para preencher lacunas na investigação sobre as relações entre infodemia e rastreio para depressão em amostras de populações idosas. Os mediadores identificados aqui podem ser abordados por intervenções psicológicas para prevenir agravamentos na saúde mental das pessoas idosas quando estas se expõem ao excesso informacional. Além disso, pode auxiliar na proposta de ações de enfrentamento e otimização de estudos que compreendam e analisem os aspectos cognitivos e comportamentais sobre o consumo de informação na terceira idade, contemplando essa temática na *práxis* da assistência psicossocial.

# Translation: Asociación entre infodemia de COVID-19 y detección de síntomas depresivos en personas mayores*

**DOI:** 10.1590/1518-8345.7454.4460

**Authors:** Patricia Rodrigues Braz, Tiago Ricardo Moreira, Andréia Queiroz Ribeiro, Rosimere Ferreira Santana, Jack Roberto Silva Fhon, Alexandre Favero Bulgarelli, Vilanice Alves de Araújo Püschel, Edna Aparecida Barbosa de Castro, Denise Rocha Raimundo Leone, Ricardo Bezerra Cavalcante

**Affiliations:** 1 Universidade Federal de Juiz de Fora, Juiz de Fora, MG, Brasil; 2 Universidade Federal de Viçosa, Viçosa, MG, Brasil; 3 Universidade Federal Fluminense, Niterói, RJ, Brasil; 4 Universidade de São Paulo, Escola de Enfermagem, São Paulo, SP, Brasil; 5 Universidade Federal do Rio Grande do Sul, Porto Alegre, RS, Brasil

**Keywords:** Infodemia, COVID-19, Salud del Anciano, Salud Mental, Depresión, Trastorno Depresivo

## Abstract

analizar el perfil de exposición a la información sobre COVID-19 y su asociación con la detección de síntomas depresivos en una muestra de adultos mayores en Brasil.

estudio transversal con datos recogidos por *web-based survey* con 3.307 participantes reclutados por medio de las redes sociales y de correo electrónico. Se realizó un análisis descriptivo, bivariado, para estimar asociaciones de interés y regresión logística cruda y ajustada por variables predictoras, sociodemográficas e infodémicas.

se encontró una asociación significativa entre el resultado de la detección de síntomas depresivos entre los adultos mayores que estuvieron expuestos durante tres a seis horas o más a las redes sociales y a la televisión y entre aquellos que informaron no haber estado expuestos a noticias e información sobre COVID-19 en la televisión.

las mujeres mayores expuestas frecuentemente a información relacionada con COVID-19 en la televisión y en las redes sociales, por periodos de entre dos y seis horas, presentaron síntomas depresivos. Este estudio presenta contribuciones para la investigación sobre infodemia y salud mental, una vez que aborda un vacío de investigación sobre la relación entre la detección de síntomas depresivos en una muestra de personas mayores y su asociación con el perfil de exposición a la información sobre COVID-19.

## Destacados

**(1)** Existe una asociación entre las variables infodémicas y la detección de la depresión **(2)** Originalidad en la investigación sobre los impactos de la infodemia en la detección de la depresión. **(3)** Los adultos mayores que no se expusieron a las noticias también se sometieron a detección de la depresión. **(4)** El estudio presenta contribuciones a la investigación sobre infodemia y salud mental.

## Introducción

La COVID-19 se consideró un nuevo tipo de enfermedad viral y, en el período de su brote, el escenario era incipiente respecto a la información científica y proveniente de instituciones gubernamentales sobre la enfermedad. En ese contexto, los medios de comunicación potenciaron la difusión excesiva de información. Este proceso se caracterizó como una infodemia, término que se refiere al exceso de información asociada a un tema específico, que puede multiplicarse exponencialmente en poco tiempo debido a un evento concreto^(1-2)^, y que también favorece la aparición de información errónea, desinformación, además de la manipulación de información con dudosa intención^(2)^. La infodemia de COVID-19 fue ampliamente reconocida por haber agravado el contexto de la pandemia, configurándose como un fenómeno que potencia la difusión de contenidos inadecuados^(3)^.

Los adultos mayores, especialmente en Brasil, pueden ser más vulnerables a la desinformación y, en consecuencia, contribuir a la difusión de noticias falsas. No necesariamente por la edad, sino por algunas características que suelen ser más comunes en esta etapa de la vida.

Hay un conjunto de características que parecen hacer que los adultos mayores en Brasil sean más vulnerables al consumo y a la difusión de noticias falsas sobre COVID-19. El analfabetismo funcional es un factor interviniente. En Brasil, la última encuesta realizada sobre analfabetismo funcional en el 2018 mostró que el 10,3% del grupo de población de 60 años o más se clasificaba como analfabetos funcionales^(4)^. También hay deficiencias en la alfabetización sanitaria y digital, agregados a la escasa alfabetización mediática y la mínima alfabetización científica^(5-7)^.

Esta coyuntura infodémica ha tenido repercusiones negativas en la salud mental de la población adulta mayor. Estudios^(8-13)^ realizados con muestras de adultos identificaron un aumento de casos de depresión asociados al exceso de información, así como recurrencias de casos de depresión causada por información negativa sobre COVID-19^(13-14)^. Además, existen evidencias de que individuos deprimidos tienden a usar con frecuencia las redes sociales^(15)^.

Durante la pandemia de COVID-19, el diagnóstico de depresión aumentó en todo el mundo^(16-18)^. En el primer año de la pandemia, la prevalencia mundial de la depresión aumentó un 25%. En 2020, antes de la pandemia, alrededor de 193 millones de personas padecían un trastorno depresivo mayor, pero tras el advenimiento de la pandemia, las estimaciones muestran un salto hasta los 246 millones por trastorno depresivo mayor, lo que representa un aumento del 28% en el año 2021^(17)^. Actualmente, en América Latina, Brasil es el país con mayor prevalencia de depresión, además de ser el segundo país con mayor prevalencia en las Américas^(18)^, con mayor prevalencia de la enfermedad en la población adulta mayor^(19)^.

Los criterios de diagnóstico de un episodio depresivo según la Clasificación Internacional de Enfermedades (CIE-10) incluyen abatimiento del ánimo, reducción de la energía, disminución de la actividad, alteración de la capacidad para experimentar placer, pérdida de interés, disminución de la capacidad de concentración, asociada con fatiga. En un episodio depresivo leve, al menos dos o tres de estos síntomas generalmente están presentes, mientras que en un episodio depresivo severo sin síntomas psicóticos, varios de los síntomas son perceptibles y angustiantes, e implican pérdida de autoestima y sentimientos de culpa. Además, las ideas suicidas son comunes y generalmente se observa una serie de síntomas somáticos^(20)^.

En vista de lo anterior, estudios demuestran que la infodemia de COVID-19 tuvo un impacto negativo en la salud mental de los adultos mayores, por lo que resulta relevante investigar dicho fenómeno y sus repercusiones en la salud mental de la población adulta mayor en cuanto a la detección de síntomas depresivos. Las investigaciones en este ámbito con muestras de población adulta mayor son aún escasas. Esta investigación tuvo como objetivo analizar el perfil de exposición a información sobre COVID-19 y su asociación con la detección de síntomas depresivos en una muestra de adultos mayores en Brasil.

## Método

### Contexto y período

Se trata de un estudio descriptivo, exploratorio y transversal realizado con adultos mayores en Brasil. La recopilación de datos se llevó a cabo entre julio del 2020 y marzo del 2021.

### Muestra y criterios de selección

El tamaño de la muestra del estudio multicéntrico fue estimado por municipio, considerando la población de adultos mayores local, mediante la fórmula: n= N. Z².p.(1−p) /Z².p.(1−p). + e². (N-1), donde “n” es el tamaño de la muestra calculado, “N” es el tamaño de la población, “Z”, la variable normal estandarizada asociada al nivel de confianza (en este caso, 95%), “p”, la verdadera probabilidad del evento (P= (1−P) =0,5, supuesto de máxima probabilidad), y “e”, el error muestral, utilizando un error muestral del 5%. La muestra para datos parciales consideró: 50% de prevalencia del fenómeno, 3% de error, 95% de nivel de confianza + 10% de análisis múltiples y 20% considerando posibles pérdidas dentro de la muestra (respuestas en blanco). En Brasil el muestreo fue no probabilístico. La base de datos contó inicialmente con 3.321 participantes, se excluyeron duplicidades de información y participantes que no completaron toda la información, en total participaron en la investigación 3.307 adultos mayores brasileños.

Los participantes elegibles para este estudio fueron adultos mayores brasileños de 60 años o más, que tenían acceso a redes sociales y correo electrónico o teléfono, y capacidad para responder el cuestionario por medio de las redes sociales o por teléfono. El criterio de exclusión fue declarar que no tenían habilidades para responder el cuestionario a través de medios digitales o incluso por teléfono, así como los adultos mayores que viven en Instituciones de Larga Estancia para Adultos Mayores (ILPI, por sus siglas en portugués), entendiendo que el proceso de la institucionalización de los adultos mayores en sí mismo solo puede contribuir al desarrollo o empeoramiento de cuadros depresivos y de ansiedad^(21)^.

Se utilizaron diferentes estrategias para asegurar una mayor representatividad de la muestra, se utilizó la estrategia de bola de nieve virtual, compartiendo el enlace para participar en la investigación con sociedades científicas de geriatría y gerontología, unidades asistenciales de salud, asociaciones de jubilados y, de manera directa, las personas mayores que ya recibían seguimiento mediante actividades de investigación y extensión en los centros colaboradores de la investigación.

### Instrumentos utilizados para recopilar información y variables del estudio

El instrumento de recopilación de datos sobre exposición a información sobre COVID-19 fue adaptado de estudios previos sobre infodemia^(8,22)^. Además de las variables socioeconómicas, hubo variables relacionadas con la infodemia sobre COVID-19 en las redes sociales, radio y televisión que hicieron referencia al tipo de medio al que se accedió y al tiempo de exposición a los medios (frecuencia y horas).

Para evaluar la detección de síntomas depresivos se utilizó la Escala de Depresión Geriátrica (EDG). Se adoptó el punto de corte 5/6 (no caso/caso), que se considera apropiado para detectar, identificar tempranamente y categorizar síntomas depresivos en adultos mayores en entornos no especializados en Brasil^(23)^.

### Recopilación de datos

El instrumento de recopilación de datos se estructuró mediante una *web-based survey*. Al acceder por primera vez al enlace, las personas mayores eran dirigidas al Consentimiento Libre, Previo e Informado (CLPI) digital para que lo leyeran y eligieran aceptar o negarse a participar en el estudio. La opción de participar o no en el estudio quedó registrada automáticamente en la base de datos generada por la *web-based survey.* Quienes optaron por continuar participando en el estudio tuvieron acceso a las preguntas de investigación.

### Tratamiento y análisis de datos

Los procedimientos de tratamiento y análisis se llevaron a cabo por medio del programa. *Statistical Package for the Social Sciences* ^®^ (SPSS) versión 23.0. Se realizaron análisis descriptivos mediante la caracterización demográfica de los participantes y de las variables relativas a la exposición a noticias e información relacionadas con COVID-19 en los medios de comunicación. En el caso de las variables cualitativas, se estimaron frecuencias absolutas y relativas. En el caso de las variables cuantitativas, se calcularon medidas de posición (media y mediana) y dispersión (desviación estándar, rango intercuartil, mínimo y máximo), dependiendo de la distribución de los datos (simétrica o asimétrica).

La asociación entre las variables infodémicas y los desenlaces relacionados con la detección de síntomas depresivos se evaluó mediante la prueba de chi-cuadrado. A continuación, se realizó un análisis de regresión logística múltiple crudo y ajustado (por sexo, rango de edad, educación, convivencia y cambios en los ingresos durante la pandemia) para la asociación entre las variables independientes de interés y el desenlace de depresión, con respectivos intervalos de confianza del 95%. El nivel de significancia adoptado en todas las pruebas fue del 5%.

### Aspectos éticos

El estudio fue aprobado por la Comisión Nacional de Ética en Investigación (Conep) de Brasil el 03/07/2020 – Certificado de Presentación de Apreciación Ética (CAAE): 31932620.1.1001.5147, bajo el dictamen n.º 4.134.050, y, tras su aprobación, se inició la recopilación de datos. Está previsto el cumplimiento de todos los requisitos éticos y legales propios de las investigaciones con seres humanos, de acuerdo con las disposiciones reglamentarias de la Resolución brasileña n.º 466/12 del Consejo Nacional de Salud.

A los entrevistados se les aseguró el anonimato, mediante el uso de números para representarlos en el estudio, la aclaración del objetivo, justificación y procedimientos de la investigación, así como la explicación respecto a la participación voluntaria, sin ventajas económicas ni gastos y sobre la divulgación de los hallazgos, que únicamente se producirá en eventos o revistas de carácter científico.

## Resultados

Participaron en el estudio 3.307 adultos mayores, el 68,4% eran mujeres, el 38,9% tenían entre 60 y 64 años, el 55,5% estaban casados y el 71,5% se autodeclaraban blancos. En cuanto a la vivienda, el 95,6% vivía en zona urbana y el 57,0% vivía con una o dos personas en su hogar. De los participantes, solo el 8,9% no estudió o no completó la educación básica, mientras que el 19,5% había completado la educación superior. La mayoría, el 40,6%, utilizaba servicios de salud tanto gratuitos como de pago. Y el 73,8% de los participantes respondió que la pandemia no afectó sus ingresos mensuales.

En cuanto a la detección de síntomas depresivos relacionados con variables sociodemográficas, se destaca que en el 66,7% de los adultos mayores que prefirió no declarar su sexo se detectaron síntomas depresivos, en el 44,7% de los adultos mayores más longevos, de 80 años o más, se detectaron síntomas depresivos, y este fue el subgrupo de edad con el mayor porcentaje. En cuanto al estado civil, en el 46,3% de los adultos mayores solteros se detectaron síntomas depresivos. Con respecto a la convivencia, en el 37,3% de las personas mayores que vivía sola se detectaron síntomas depresivos, y, en cuanto a la educación y años de estudio, en el 47,1% de las personas mayores que no tuvieron la oportunidad de estudiar o completar la educación básica se detectaron síntomas depresivos (Tabla 1).

**Tabla 1 -:** Perfil demográfico y de detección de síntomas depresivos en adultos mayores que participaron en la investigación (n = 3.307). Brasil, 2021

**Variables**	**N**	**%**	**Detección de síntomas depresivos**			
			**No caso**		**Caso**	
			**N**	**%**	**N**	**%**
**Sexo**						
Femenino	2.250	68,4	1.347	59,9%	903	40,1%
Masculino	1.039	31,6	638	61,4%	401	38,6%
Prefiero no responder	18	0,5	6	33,3%	12	66,7%
**Rango de edad (años)**						
60 a 64	1.285	38,9	799	62,2%	486	37,8%
65 a 69	921	27,9	543	59,0%	378	41,0%
70 a 74	503	15,2	309	61,4%	194	38,6%
75 a 79	334	10,1	194	58,1%	140	41,9%
80 o más	264	8,0	146	55,3%	118	44,7%
**Estado civil:**						
Casado(a)/viviendo juntos	1.835	55,5	1.119	61,0%	716	39,0%
Viudo(a)	598	18,1	340	56,9%	258	43,1%
Separado(a)/divorciado(a)	509	15,4	336	66,0%	173	34,0%
Soltero(a)	365	11,0	196	53,7%	169	46,3%
**Raza/color**						
Blanco	2.364	71,5	1.436	60,7%	928	39,3%
No blanco	943	28,5	555	58,9%	388	41,1%
**Cohabitación**						
Vive solo(a)	587	17,8	368	62,7%	219	37,3%
Una o dos personas	1.886	57,0	1.129	59,9%	757	40,1%
Tres o más personas	834	25,2	494	59,2%	340	40,8%
**Residencia propia**						
Sí	2.756	83,3	1.668	60,5%	1.088	39,5%
No	551	16,7	323	58,6%	228	41,4%
**Zona de residencia**						
Zona urbana	3.160	95,6	1.910	60,4%	1.250	39,6%
Zona rural	147	4,4	81	55,1%	66	44,9%
**Escolaridad máxima**						
No estudió o no completó la educación básica	295	8,9	156	52,9%	139	47,1%
Educación básica o primaria	713	21,6	409	57,4%	304	42,6%
Educación secundaria	718	21,7	449	62,5%	269	37,5%
Educación superior completa	645	19,5	389	60,3%	256	39,7%
Especialización	512	15,5	322	62,9%	190	37,1%
Máster o doctorado	424	12,8	266	62,7%	158	37,3%
**Servicios de salud que utiliza**						
Solo servicios sanitarios de pago (incluido el seguro médico)	1.133	34,3	692	61,1%	441	38,9%
Ambos (gratuitos y de pago)	1.343	40,6	823	61,3%	520	38,7%
Solo servicios de salud gratuitos	814	24,6	466	57,2%	348	42,8%
Ninguno	17	0,5	10	58,8%	7	41,2%
**Cobra jubilación o pensión**						
Sí	2.565	77,6	1.520	59,3%	1.045	40,7%
No	740	22,40	470	63,5%	270	36,5%
**La pandemia cambió sus ingresos**						
No	2.437	73,8	1.469	60,3%	968	39,7%
Sí, mis ingresos han disminuido	787	23,8	47	58,8%	33	41,2%
Sí, mis ingresos han aumentado	80	2,4	474	60,2%	313	39,8%

Respecto a los recursos más utilizados para acceder a noticias e información sobre COVID-19 durante el día, el 81,1% de los adultos mayores utilizó la televisión, el 58,8% utilizó las redes sociales y solo el 26,48% utilizó la radio. Los adultos mayores también informaron su percepción de exposición a información y noticias sobre COVID-19 durante un período de una semana (siete días), variando desde respuestas de “ninguna exposición” hasta respuestas de “exposición frecuente”. La televisión fue reportada como una fuente de exposición “frecuente” (44,5%), seguida por las redes sociales “a veces” (44,3%). En cambio, la radio no representó una fuente de exposición para la mayoría de los adultos mayores (59,1%). En cuanto a las horas de exposición, el 39,3% de los adultos mayores estuvo expuesto a la televisión durante tres horas o más, el 32,8% accedió a las redes sociales entre dos y cinco horas, y el 37,0% estuvo expuesta a la radio durante una hora o más (Tabla 2).

Se encontró una asociación significativa (*P* > 0,05, es decir, probabilidad superior al 5%) entre el resultado de la detección de presencia de síntomas depresivos y las variables frecuencia de exposición a la televisión (*P* = 0,01), horas de exposición a noticias e información sobre COVID-19 en las redes sociales (*P* =0,00 ) y en la televisión (*P* =0,02). Respecto a la variable frecuencia de exposición a la televisión, en el 42,9% de los adultos mayores que declararon no exponerse a la información se detectaron síntomas depresivos. En cuanto a las horas de exposición, en el 44,3% de los adultos mayores que declararon haber estado expuestos durante seis horas o más a noticias e información sobre COVID-19 en las redes sociales se detectó la presencia de síntomas depresivos. Y en el 43,2% que informó haber estado expuesto a noticias e información sobre COVID-19 en la televisión durante tres horas o más también se detectó la presencia de síntomas depresivos (Tabla 3).

**Tabla 2 -:** Caracterización de los medios de información más utilizados para acceder a noticias e información sobre COVID-19 por los adultos mayores participantes (n = 3.307). Brasil, 2021

**Medios de información**	**N**	**%**
**Redes sociales (N=3.303)**		
Sí	1.943	58,8
No	1.361	41,2
**Frecuencia de exposición**		
Ninguna	822	24,9
A veces	1.464	44,3
Frecuentemente	1.021	30,9
**Horas de exposición a noticias e información sobre COVID-19 en redes sociales**		
Ninguna	848	25,6
Una	811	24,5
Dos a cinco	1.084	32,8
Seis o más	560	16,9
**Total (n=3.304)**		
Sí	2.680	81,1
No	624	18,9
**Frecuencia de exposición**		
Ninguna	394	11,9
A veces	1.440	43,5
Frecuentemente	1.473	44,5
**Horas de exposición a noticias e información sobre el COVID-19 en televisión**		
Ninguna	431	13,0
Una	884	26,7
Dos	685	20,7
Tres o más	1.301	39,3
**Radio (n=3.304)**		
Sí	876	26,5
No	2.429	73,5
**Frecuencia de exposición**		
Ninguna	1.956	59,1
A veces	956	28,9
Frecuentemente	395	11,9
**Horas de exposición a noticias e información sobre COVID-19 en la radio**		
Ninguna	2.083	63,0
Uno o más	1.223	37,0

**Tabla 3 -:** Asociación entre variables infodémicas y la detección de síntomas depresivos entre los adultos mayores participantes (n = 3.307). Brasil, 2021

**Variable**	**Presencia de síntomas depresivos**				**Valor de *P***
	**No caso**		**Caso**		
	**N**	**%**	**N**	**%**	
**Frecuencia de exposición a las redes sociales.**					0,08
Ninguna	463	56,3%	359	43,7%	
A veces	920	62,8%	544	37,2%	
Frecuentemente	608	59,5%	413	40,5%	
**Frecuencia de exposición a la televisión**					0,01
Ninguna	225	57,1%	169	42,9%	
A veces	909	63,1%	531	36,9%	
Frecuentemente	857	58,2%	616	41,8%	
**Frecuencia de exposición a la radio**					0,07
Ninguna	1182	60,4%	774	39,6%	
A veces	591	61,8%	365	38,2%	
Frecuentemente	218	55,2%	177	44,8%	
**Horas de exposición a noticias e información sobre COVID-19 en las redes sociales**					0,00
Ninguna	482	56,8%	366	43,2%	
Una	529	65,2%	282	34,8%	
Dos a cinco	665	61,3%	419	38,7%	
Seis o más	312	55,7%	248	44,3%	
**Horas de exposición a noticias e información sobre COVID-19 en la televisión**					0,02
Ninguna	254	58,9%	177	41,1%	
Una	573	64,8%	311	35,2%	
Dos	421	61,5%	264	38,5%	
Tres o más	739	56,8%	562	43,2%	
**Horas de exposición a noticias e información sobre COVID-19 en la radio**					0,83
Ninguna	1251	60,1%	832	39,9%	
Una o más	739	60,4%	484	39,6%	

El análisis demostró que, en la regresión logística cruda, estaban asociados con la detección de síntomas depresivos estar expuesto frecuentemente a noticias e información sobre COVID-19 en la radio (*P* =0,02) y la televisión (*P* =0,00), así como estar expuesto durante dos horas (*P* =0,00) o durante tres horas o más (*P* = 0,04) a la televisión y estar expuesto de dos a cinco horas (*P* = 0,00) o durante seis horas o más (*P* =0,02) a las redes sociales. En el análisis ajustado, las variables infodémicas que permanecieron asociadas con la detección de síntomas depresivos (*P* ≤0,05) fueron la exposición frecuente a televisión y radio, la exposición de dos a cinco horas, o de seis horas o más a las redes sociales, y la exposición a televisión durante dos horas (Tabla 4).

**Tabla 4 -:** Modelo de Regresión Logística cruda y ajustada por la suma de la Escala de Depresión Geriátrica aplicada a los adultos mayores participantes (n = 3.307). Brasil, 2021

**Presencia de síntomas depresivos (regresión logística)**		
	Análisis crudo OR(IC95%)*	Análisis ajustado OR(IC95%)*
**Frecuencia de exposición a las redes sociales**		
Ninguna	1	1
A veces	1,14 (0,94 - 1,37)	1,01 (0,82- 1,25)
Frecuentemente	0,87 (0,73 - 1,02)	0,85 (0,72- 1,01)
**Frecuencia de exposición a la televisión**		
Ninguna	1	1
A veces	1,04 (0,83- 1,30)	1,06 (0,85- 1,34)
Frecuentemente	0,81 (0,70 -0,94)	0,81 (0,70- 0,94)
**Frecuencia de exposición a la radio**		
Ninguna	1	1
A veces	0,80 (0,64 - 1,00)	0,80 (0,64- 1,00)
Frecuentemente	0,76 (0,60 – 0,96)	0,74 (0,58- 0,95)
**Horas de exposición a noticias e información sobre COVID-19 en las redes sociales**		
Ninguna	1	1
Una	0,95 (0,77 -1,18)	0,86 (0,68- 1,09)
Dos a cinco	0,67 (0,53 -0,83)	0,68 (0,54 - 0,85)
Seis o más	0,79 (0,64 - 0,97)	0,80 (0,64- 0,98)
**Horas de exposición a noticias e información sobre COVID-19 en la televisión**		
Ninguna	1	1
Una	0,91 (0,73- 1,14)	0,95 (0,76 - 1,20)
Dos	0,71 (0,59 -0,85)	0,73 (0,61 - 0,87)
Tres o más	0,82 (0,68 - 0,99)	0,85 (0,70 - 1,02)
**Horas de exposición a noticias e información sobre COVID-19 en la radio**		
Ninguna	1	1
Una o más	1,01 (0,87 -1,17)	1,03 (0,89 - 1,20)

*OR(IC95%) = *Odds ratio* e intervalo de confianza

## Discusión

El estudio tuvo como objetivo investigar la infodemia de COVID-19 y sus repercusiones en la salud mental de los adultos mayores, en cuanto a la detección de síntomas depresivos.

Respecto a las variables infodémicas, resulta relevante la reflexión sobre el escenario temporal de la pandemia. Durante el período de desarrollo del estudio, entre julio del 2020 y marzo del 2021, el escenario epidemiológico de COVID-19 fue alarmante. En cuanto a los datos epidemiológicos, según el Ministerio de Salud^(24)^ brasileño, el promedio de muertes fue de 1.026 en el segundo semestre del 2020; el documento también muestra que el mes de marzo del 2021 tuvo el mayor porcentaje de muertes, con un promedio de 2.207 muertes al día^(24)^.

Las reglas de distanciamiento social aún estaban vigentes, lo que fomentó un mayor tiempo de consumo de medios y aumento en el hábito de ver la televisión entre la población adulta. Los medios de comunicación destacaron la información sobre el número de muertes e infectados. En ese contexto, también hubo información relacionada con la disputa política por la compra y distribución de vacunas, dado que los primeros inmunizantes solo llegaron al país en enero del 2021^(8,12)^.

Los resultados de esta investigación identificaron que la televisión fue el medio más utilizado para acceder a la información y también fue considerada la fuente de información a la que se expusieron con mayor frecuencia los adultos mayores, con una exposición que duraba tres horas o más, seguida de las redes sociales. La radio fue la fuente de información a la que menos se expusieron los adultos mayores.

Un estudio realizado en Brasil durante la pandemia de COVID-19, en el que participaron 45.161 personas, de las cuales el 20,3% eran personas mayores de 60 años o más, constató un aumento en el consumo de televisión, y los adultos mayores fueron el grupo que más tiempo se expuso a la televisión. La investigación también afirma que el 61,6% de los participantes permanecieron expuestos a este medio durante tres horas o más^(25)^.

Otra investigación analizó el comportamiento de búsqueda de información de los adultos mayores durante la pandemia de COVID-19 e identificó que la televisión fue el principal vehículo de comunicación referido por los adultos mayores^(26)^. Los adultos mayores entrevistadas afirmaron que su preferencia por la televisión se debía a la facilidad acceso, así como a la velocidad con la que obtuvieron la información, mediante actualizaciones sobre los acontecimientos diarios en todo el mundo^(27)^.

En el caso de los adultos mayores, la televisión sigue siendo el medio más sencillo, rápido y cómodo de acceder a la información^(3,12,26)^, una vez que pueden tener mayores dificultades para usar y manipular computadoras y teléfonos celulares.

Otra investigación realizada con 799 participantes adultos en Rio Grande do Sul constató que el medio más utilizado por los participantes para acceder a información sobre COVID-19 fue la televisión (76,3%) seguido de las redes sociales, más precisamente mediante el uso de *Facebook* (37%), *WhatsApp* (28,3%), *Instagram* (25,8%), *Twitter* (20,7%) entre otras. Y el medio menos utilizado fue la radio (9%)^(12)^.

La urgencia de información y su difusión generalizada sobre la pandemia de COVID-19 se asoció con sentimientos de depresión y miedo^(11,13,16)^. Los resultados obtenidos en este estudio confirman las evidencias científicas, especialmente en lo que respecta a los adultos mayores. En los adultos mayores que estuvieron expuestos con frecuencia a noticias e información sobre COVID-19 por medio de la televisión y las redes sociales, además de usar estos medios durante entre dos y seis horas o más, se detectaron síntomas de depresión con mayor frecuencia.

Los investigadores de un estudio realizado con 1.577 adultos comunitarios y 214 profesionales sanitarios investigaron los factores de riesgo de depresión en personas de la comunidad y entre profesionales de la salud en el epicentro de Wuhan, China, y se demostró que estar expuesto a noticias sobre COVID-19 por medio de las redes sociales durante dos horas al día o más se asociaba con este trastorno en adultos (37,16% n=586). El estudio advierte que las redes sociales pueden desempeñar un papel específico en el sufrimiento mental de la población durante epidemias^(16)^. Otra investigación realizada en Singapur con 1.145 adultos demostró que las puntuaciones de depresión estaban relacionadas positivamente con un mayor tiempo dedicado a recibir actualizaciones sobre COVID-19 en las redes sociales y en la televisión^(13)^.

Una revisión de alcance analizó 33 artículos sobre infodemia y salud mental de adultos y adultos mayores, enfatizando los signos y síntomas más mencionados en las publicaciones analizadas. Se observó que la depresión aparece en un 51,5% de las publicaciones. Este resultado refuerza la idea de que la exposición excesiva a la información está directamente relacionada con el desarrollo de problemas de salud mental^(11)^.

Si bien existe una tendencia en la literatura científica a atribuir los síntomas de la depresión al uso de las Tecnologías de la Información y la Comunicación (TIC), es necesario considerar la causalidad inversa. En este caso, se considera que la depresión puede llevar a la rumiación en las redes sociales en busca de información, como ocupación del tiempo libre o por distracción^(16,28-29)^. Una encuesta realizada en Canadá y Estados Unidos a 685 adultos encontró que el uso de las redes sociales y la televisión fue mayor entre los 621 encuestados que declararon que tenían miedo, estaban preocupados y presentaban otros síntomas de sufrimiento mental. Los encuestados que consideraban que su salud mental era “mala” tenían el doble de probabilidades de optar por utilizar computadoras y teléfonos celulares como herramientas para afrontar el autoaislamiento^(28)^.

La necesidad de información del usuario puede estar relacionada con el “vacío cognitivo”. La percepción e interpretación de este “vacío”, caracterizado por la incertidumbre y la necesidad de buscar, modula el comportamiento del individuo a la hora de acceder a la información, es decir, existe el deseo de llenar el vacío existente y, así, intentar superar esa “ausencia”^(30)^.

Una reseña sobre el impacto de las redes sociales en la percepción de soledad o en el aislamiento social en adultos mayores concluyó que las evidencias científicas han demostrado que el uso de las redes sociales digitales puede reducir la percepción de soledad o aislamiento en adultos mayores, puede favorecer un mayor contacto entre los adultos mayores y las familias, servir como fuente de apoyo, contribuir a una mayor sensación de control sobre sus vidas y a un mayor sentido de pertenencia a la comunidad. El estudio resalta que internet puede considerarse importante para reducir las barreras del aislamiento^(31)^. Sin embargo, en un escenario infodémico, la búsqueda constante de llenar vacíos concluyentes sobre los problemas puede terminar generando desinformación, la mala información y la información errónea.

En los estudios contemporáneos sobre la “sociedad del desempeño”, se resalta que esta sociedad está estructurada en gran medida por el consumo de información. En la sociedad del desempeño, mantenida en la cultura digital, prevalece la retórica del querer es poder. Esta premisa plantea la idea de que, actualmente, solo no tiene acceso a la información sobre un acontecimiento en el mundo quien no quiere. Para los adultos mayores, comprender este nuevo modelo informativo puede provocar conflictos internos sobre el ser y estar en el lugar social como consumidores de información y mediadores de comunicación. Por lo tanto, están incluidos, aunque no sea intencionalmente, en esta estructura^(32-33)^.

Sin embargo, la presente investigación demostró que en los adultos mayores que relataron no exponerse a información en las redes sociales y la televisión también se detectaron síntomas de depresión. La evasión de la información puede llevar a las personas a la percepción de que se están perdiendo información nueva importante, hecho que puede generar estrés y ansiedad, y es una actitud de afrontamiento mal adaptativa^(33)^.

Una encuesta realizada con 1.059 alemanes a quienes se les pidió que informaran sobre sus emociones y comportamiento desde el inicio de la pandemia de COVID-19 dio como resultado la comprensión de que la opción de evitar la información puede estar relacionada con la sensación de que no se puede hacer nada para evitar las consecuencias negativas de la enfermedad. Los participantes que estaban “muy preocupados” por los riesgos para la salud mental y física también tenían más probabilidades de evitar o ignorar a los medios de comunicación. El análisis también reveló que un mayor nivel de estrés, ansiedad y depresión se asociaba con un menor acceso a las redes sociales^(34)^.

Una limitación del estudio se refiere al uso de *web-based survey* para recopilar datos, lo que, en consecuencia, interfirió negativamente en la participación en la investigación de una porción importante de adultos mayores que no tienen acceso a internet ni a las redes sociales, lo que culminó en un sesgo muestral.

El estudio aporta contribuciones y avances a la práctica, la investigación y sus consecuencias políticas. En la práctica, los resultados resaltan la necesidad de intervenciones de enfermería frente a las repercusiones de la infodemia en la salud mental de las personas mayores y dan lugar a la necesidad de desarrollar estrategias de educación sanitaria con relación a la infodemia adecuadas para los adultos mayores por medio de la difusión de información clara y objetiva, adecuada y necesaria, encaminada a una mejor calidad de vida.

En la investigación, los resultados resaltan la importancia de promover y aplicar herramientas para la gestión de infodemias, con el objetivo de monitorear sus impactos en escenarios de emergencia sanitaria en el ámbito de la comunicación. La gestión del exceso de información debe ser un tema de debate en la salud pública, dado que el fenómeno infodémico tiene su complejidad porque está centralizado en un contexto multifactorial, atravesado por influyentes cuestiones políticas y socioculturales.

## Conclusión

Se concluyó que el perfil demográfico de la muestra investigada estuvo conformado, en su mayoría, por mujeres adultas mayores, de 60 a 64 años, blancas, con alto nivel educativo, que viven con una o dos personas en su propia residencia, jubiladas y sin variación de ingresos debido a la pandemia. Se verificó la asociación entre la exposición a noticias e información sobre COVID-19 y la presencia de síntomas depresivos en las mujeres adultas mayores expuestas frecuentemente a información relacionada con COVID-19 en la televisión y las redes sociales, por períodos entre dos y seis horas. La infodemia en un contexto de pandemia en un escenario de emergencia sanitaria puede tener un impacto negativo en la salud mental de los adultos mayores.

Este estudio presenta contribuciones para llenar vacíos en la investigación sobre las relaciones entre la infodemia y la detección de síntomas de depresión en muestras de poblaciones de edad avanzada. Los mediadores identificados en este estudio pueden abordarse mediante intervenciones psicológicas para prevenir el empeoramiento de la salud mental de los adultos mayores cuando están expuestos a información excesiva. Además, puede ayudar a proponer acciones para afrontar y optimizar estudios que comprendan y analicen los aspectos cognitivos y conductuales sobre el consumo de información en la vejez, abarcando este tema en la *praxis* de la asistencia psicosocial.
